# Haemorrhage following transoral robotic surgery in head and neck cancer

**DOI:** 10.1007/s11701-025-03018-5

**Published:** 2025-12-29

**Authors:** Charles Pinhorn, Robbie Stewart, Thomas Payne, Daniel Edwards, Yuvraj Singh-Dehal, Mughilan Muralitharan, Nashreen Oozeer, David Walker, Tom Vauterin, Aleix Rovira, Jean-Pierre Jeannon, Asit Arora

**Affiliations:** 1 King’s College London, London, United Kingdom; 2 Guy’s and St Thomas’ NHS Foundation Trust, London, United Kingdom; 3 South Tyneside and Sunderland NHS Foundation Trust, South Shields, United Kingdom; 4 Royal Surrey County Hospital, Guildford, United Kingdom; 5 AZ Sint-Jan Brugge AV, Bruges, Belgium

**Keywords:** Transoral robotic surgery (TORS), Haemostasis, Haemorrhage, Bleeding, Head and neck

## Abstract

As the utilisation of transoral robotic surgery (TORS) continues to rise, there is a growing need to evaluate the risks of perioperative complications. Post-TORS haemorrhage represents the most severe complication associated with TORS, however currently there is no consensus for managing these patients. This review assessed the extent of post-TORS haemorrhage in head and neck cancer (HNC) patients and evaluated current approaches for achieving haemostasis following post-TORS haemorrhage. A comprehensive search of Medline, Embase & Web of Science was conducted, to identify articles published from the databases’ inception to January 2025. Severity, incidence & management strategies employed in these studies were examined, in addition to risk factors associated with post-TORS haemorrhage. 28 studies met our inclusion criteria. The pooled average bleeding incidence for HNC patients following TORS was 7.24%, with the median day for initial bleeding episode occurring post-operative day 6. Classification of post-TORS haemorrhage severity was documented for 28.5% of reported bleeds, highlighting the need to adopt a classification system. Management strategies for achieving haemostasis varied significantly between institutions, and granularity with respect to airway management was poorly reported. Currently, consensus regarding an appropriate stepwise approach to managing post-TORS haemorrhage remains contested, as highlighted by the varied nature of haemostatic management techniques employed across a range of institutions in the included literature. As data becomes more readily available a standardised approach to classification of haemorrhage severity will be possible and consensus on haemostatic techniques can be made, which will support the creation of best practice guidelines.

## Introduction

In recent years, there has been an increase in the incidence of head and neck cancer (HNC) globally, despite declines in traditional risk factors associated with HNC (i.e. tobacco & alcohol consumption) [1, 2]. In North America and Western Europe, this rise is largely attributable to the emergence of oropharyngeal HNC associated with human papilloma virus (HPV), specifically HPV-16 & 18 subtypes [2, 3]. By 2030 oropharyngeal cancer within the United States of America (USA) is expected to represent close to 50% of all head and neck cancers [4].

The rising number of HPV associated oropharyngeal cancers, has led to a shift in the classical patient demographic for oropharyngeal cancer. Typically, patients now present at a younger age and experience improved prognosis and survivorship following treatment [3, 5]. This had led to increasing preference for treatment modalities which focus on improved functional outcomes & quality of life.

In recent decades, the mainstay of treatment for oropharyngeal cancer has revolved around chemoradiotherapy (CRT), as this was proven to have lower morbidity compared to traditional open surgical techniques. Despite the promising oncological outcomes associated with CRT, patients are likely to suffer varying degrees of toxicity following treatment initiation, which significantly impacts quality of life [6]. The first application of robotic technology in surgery was described in 1985 when a robot was used to define the trajectory for a stereotactic brain biopsy [7]. Since the approval of TORS for the treatment of T1 and T2 head and neck malignancies in 2009, by the Food and Drug Administration (FDA), this minimally invasive surgical modality has emerged as a safe and effective alternative treatment modality, with good oncological and functional outcomes for patients [8–14]. In addition to its therapeutic role, TORS contributes to the diagnostic pathway for head and neck cancers of unknown primary origin (CUP) [15]. The identification of which can allow for reduction in radiotherapy fields and improved functional outcomes. Since its introduction it has become an important component in the management of head and neck cancer in the United Kingdom [16].

Although TORS is associated with more preferrable functional outcomes and financial implications than traditional therapies, there are serious & potentially life threatening complications associated with this minimally invasive form of surgery [17]. Most notably, haemorrhage and the potential risk of subsequent airway compromise in the postoperative period following TORS [18, 19]. These risks form an important part of the informed consent process with the patient [20]. Currently, consensus regarding an appropriate stepwise approach for the management of patients following post-TORS haemorrhage remains contested. As TORS continues to solidify its place as the standard of care for the diagnosis of cancers of unknown primaries and treatment of oropharyngeal cancer, there is a growing need for a clear approach for managing bleeding events; especially considering in an emergency setting management may be delivered by ear nose and throat surgeons (ENT) or allied healthcare professionals who are unfamiliar with TORS. The authors hope this body of work may provide a summary of the current literature and serve as a basis to guide further research.

## Methods

This review was conducted in accordance with the 2020 PRISMA guidelines (Preferred Reporting Items for Systematic Reviews & Meta-analyses) [21]. The protocol for this review was prospectively registered via PROSPERO (CRD420250654129).

### Literature search

The following databases were searched, from inception to January 2025 for the purpose of this review: Medline (OVID), Embase (OVID) & Web of Science Core Collection. The final search was completed on the 20th of January 2025. Currently registered studies were reviewed using the following sources: WHO ICTRP registry (international clinical trials registry platform) & the ISRCTN registry (international Standard Randomised Controlled Trial Number). Both the reference list of the identified articles, and papers citing these studies were assessed, to identify any additional studies that may have been pertinent to this review.

Three reviewers (CP, YSD & MM) independently performed abstract & title screening to identify eligible papers for full text screening. Any disagreements encountered during the screening process were resolved through discussion.

The complete search strategy for this review can be found in the Appendix (1); this strategy was modified for each database respectively. Our PICO framework for this review can also be found in the Appendix (2).

### Study eligibility

Studies were deemed eligible if they specified post-TORS haemorrhage rate in adult (> 18 years old) head and neck cancer patients. Only articles published after 2009, the year that TORS was approved by FDA, were included. If multiple articles were identified to have utilised the same database, then the largest and most pertinent study was included in the final analysis.

Both retrospective and prospective studies were eligible. The following were excluded: papers not reporting original data; letters to the editor; animal studies; cadaveric studies and case reports. Additionally, studies were limited to the English language.

### Outcomes

Primary outcomes for this study focused on the incidence and severity of post-TORS haemorrhage in head & neck cancer patients. Secondary outcomes aimed to identify: predisposing risk factors associated with post-TORS haemorrhage; incidence of rebleeding; bleed subsite & median day of bleeding. In addition, we will review the management approaches utilised for achieving haemostasis.

With respect to severity classification of post-TORS haemorrhage, we aimed to evaluate which classification systems were utilised across the included studies. The Mayo Clinic classification system for transoral haemorrhage, is the most widely accepted system currently, this is summarised in Table 1. We sought to analyse the Mayo systems use across different centres and whether alternative classification systems were utilised.

With respect to predisposing risk factors, the following were analysed: prior irradiation, antithrombotic therapy use, advanced (T3/T4) versus early (T1/T2) disease, surgeon experience (< 50 cases versus > 50 cases) and prophylactic arterial ligation.

**Table 1:** Mayo clinic classification system for transoral haemorrhage [22]

Classification	Description
Normal	Patient noting the presence of blood-tinged mucus, flecks of blood, brown mucus, or red streaks.
Minor	Any description of bright red blood or blood clots. Resolved without operative intervention, regardless of whether physician evaluation or hospitalisation occurred.
Intermediate	Diffuse venous oozing or small arterial source bleeding resulting in operating room evaluation or intervention managed with monopolar or bipolar cautery.
Major	Brisk or copious bleeding requiring operative intervention. Managed with transoral or transcervical vessel ligation or interventional radiology (IR) embolisation.
Severe	Bleeding resulting in life-threatening medical complications such as: ♣ Hypoxia/airway compromise requiring tracheostomy ♣ Cardiopulmonary arrest ♣ Haemodynamic instability requiring blood transfusion

### Methodological appraisal

Level of evidence for the included studies was determined using the March 2009 Oxford Centre for Evidence-Based Medicine: Levels of Evidence. Included studies were assessed using an appropriate risk of bias tool. For case control/cohort studies, the Newcastle-Ottawa scale (NOS) was utilised. The institute of Health economics (IHE) case series studies quality appraisal checklist was used to determine the quality of case series. Any disagreements encountered during the assessment of bias were resolved through discussion.

### Data synthesis & analysis

Meta-analysis of risk ratios (RR) was performed using Cochrane Review Manger (RevMan: the Cochrane collaboration, Copenhagen, Denmark). A random effects model was generated using DerSimonian & Laird method. Heterogeneity was assessed using Cochran’s Q statistic and then quantified using the Higgins *I*^2^ statistic. Studies were weighted using inversive variance, meaning studies with less variance were assigned more weight in the analysis. Forest plots were also generated using Cochrane Review Manager.

## Results

### Literature search

**Fig. 1:**
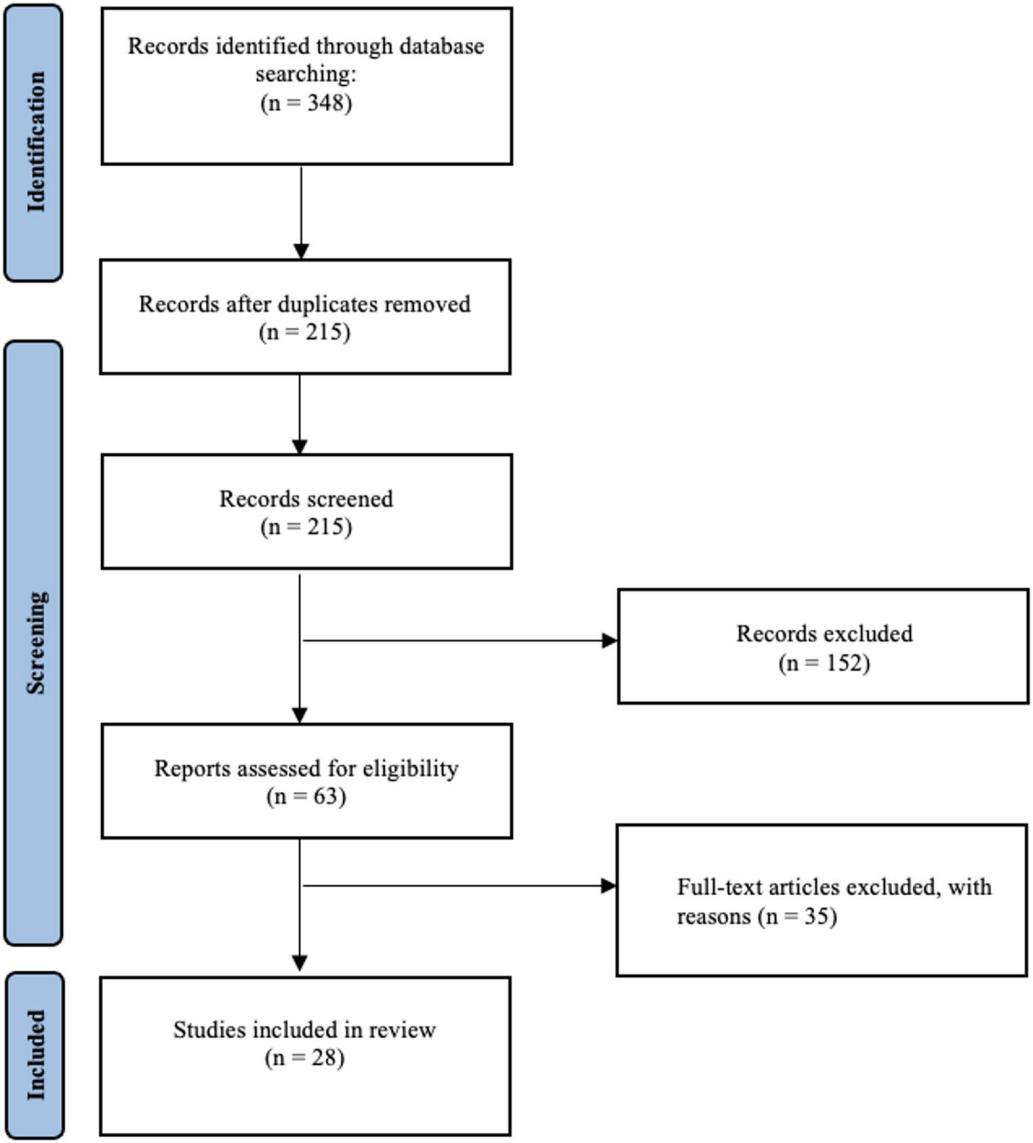
Article screening summary

Following deduplication 215 studies were screened according to eligibility criteria, as part of the title and abstract screening. In total 63 full-text articles were assessed. Ultimately 28 studies were deemed to have met the eligibility criteria and were included in this review [10, 22–48]. The screening process is detailed in Fig. 1.

### Study characteristics & patient demographics

The 28 studies included a total of 10,744 patients, of which 9852 underwent trans-oral robotic surgery for malignant indications. The median age was 60 years old, and the number of males & females included were 8498 and 2078 respectively. This data is summarised in Table 2.

**Table 2:** Study characteristics & patient demographics

Author	Publication Year	Country	Total patient number (*n*)	TORS specific number (*n*)	Malignant patient cases (%)	Patients age (years)	Male/Female
Aubry	2015	France	178	178	95%	average 61.4	141/37
Blom	2023	Australia	104	98	100%	mean 62	X
Daniels	2024	USA	221	221	100%	60.1 average	189/32
Frenkel	2017	USA	425	425	100%	129 (< 55), 149 (55–64), 108, (65–74), 39 (> 75)	336/89
Gleysteen	2017	USA	201	201	100%	median 60	169/32
Haller	2023	USA	219	219	100%	average 58.9	194/25
Hassid	2020	Belgium	22	22	100%	mean 60	18/4
Jeong	2024	Australia	55	55	100%	median 59	39/16
Kornfeld	2024	Australia	41	41	100%	average 58.1	38/3
Kubik	2017	USA	265	265	100%	mean 59	212/53
Lörincz	2014	Germany	35	35	100%	mean 65	26/9
Meulemans	2021	Belgium	61	61	100%	mean 64.5	43/18
Möckelmann	2015	Germany	41	41	100%	median 63.9 (Concurrent ND), 66.9 (staged ND)	31/10
Nyirjesy	2023	USA	5544	5544	100%	mean 60.7	4475/1069
O’Hara	2024	UK	508	313	100%	median 58.3	390/118
Olaleye	2021	Australia	49	49	100%	median 60.5	38/11
Parhar	2018	Canada	950	950	100%	mean 63.2 (readmitted), 60.9 (not readmitted)	772/178
Parhar	2021	USA	56	56	100%	median 62	40/16
Sgarzani	2019	Italy	64	64	100%	X	X
Siievert	2021	Germany	54	24	100%	mean 60.8 (TLM), 60.5 (TORS)	39/15
Porcuna	2019	Spain	54	54	100%	median 62	42/12
Winter	2017	UK	32	32	100%	mean 57	27/5
Topf	2017	USA	297	297	100%	mean 60.9%	240/57
Pollei	2013	USA	906	269	100%	average 59	752/154
Shenouda	2020	France	45	21	100%	median 61 (robotic)/62 (NR)	28/17
Parhar	2020	USA	77	77	100%	median 73	58/19
Dabas	2019	India	153	153	100%	mean 56.3	96/57
Cannavicci	2023	Italy	87	87	100%	mean 64.7+/- 9.8	65/22

### Study quality assessment

Level of evidence and risk of bias were assessed in-line with prospectively registered protocol. All studies identified were cohort studies or case series. Overall, a large proportion of the included studies were associated with methodological limitations. Findings for risk of bias (RoB), are documented in Tables 3 and 4.

**Table 3:** NOS RoB

Study	Representativeness	Selection of control	Ascertainment of exposure	Outcome of interest no present at start	Comparability outcomes	Assessment of outcome	Follow up long enough	Adequacy of follow up	Overall bias
Aubry		*	*	*		*	*	*	6/9*
Daniels	*	*	*	*		*	*	*	7/9 *
Frenkel	*	*	*	*		*	*	*	7/9 *
Gleysteen	*	*	*	*		*	*	*	7/9 *
Kubik	*	*	*	*		*	*	*	7/9 *
Möckelmann		*	*	*		*	*	*	6/9*
Nyirjesy	*	*	*	*		*	*	*	7/9 *
O’Hara	*	*	*	*		*	*	*	7/9 *
Parhar	*	*	*	*		*	*	*	7/9 *
Sgarzani	*	*	*	*		*	*	*	7/9 *
Sievert	*	*	*	*		*	*	*	7/9 *
Topf	*	*	*	*		*	*	*	7/9 *
Pollei	*	*	*	*		*	*	*	7/9*
Shenouda	*	*	*	*		*	*	*	7/9 *
Parhar	*	*	*	*		*	*	*	7/9 *

**Table 4:** IHE RoB

Study	Blom	Haller	Hassid	Jeong	Kornfeld	Lörincz	Meulemans	Olaleye	Parhar	Porcuna	Winter	Dabas	Cannavicci
Was the hypothesis/aim/objective of the study clearly stated?	Y	Y	Y	Y	Y	Y	Y	Y	Y	Y	Y	Y	Y
Was the study conducted prospectively?	N	N	N	N	N	Y	Y	Y	N	Y	Y	Y	N
Were the cases collected in more than one centre?	Y	U	N	N	N	N	N	N	Y	N	Y	U	N
Were patients recruited consecutively?	Y	U	Y	Y	U	Y	Y	Y	U	Y	Y	U	Y
Were the characteristics of the patients included in the study described?	Y	Y	Y	Y	Y	Y	Y	Y	Y	Y	N	Y	Y
Were the eligibility criteria for entry into the study clearly stated?	Y	P	Y	Y	Y	Y	Y	Y	Y	Y	Y	Y	Y
Did patients enter the study at a similar point In the disease?	Y	Y	Y	N	N	N	N	N	Y	N	N	N	N
Was the intervention of interest clearly stated?	Y	Y	Y	Y	Y	Y	Y	Y	Y	Y	Y	Y	Y
Were additional interventions clearly described?	N	N	Y	Y	Y	Y	Y	N	N	N	N	N	N
Were relevant outcomes measures established a priori?	Y	U	U	Y	Y	Y	Y	Y	U	Y	U	Y	Y
Were outcome assessors blinded to the intervention the patient received?	U	U	U	U	U	U	U	U	U	U	U	U	U
Were the relevant outcome es measured using appropriate objective/subjective methods	Y	Y	Y	Y	Y	Y	Y	Y	Y	Y	Y	Y	Y
Were the statistical tests used to assess the relevant outcomes appropriate?	NA	Y	Y	Y	Y	NA	Y	NA	Y	Y	NA	NA	Y
Was follow up long enough for important events & outcomes to occur?	U	Y	Y	Y	Y	Y	Y	U	Y	Y	U	Y	Y
Were losses to follow up reported?	N	N	N	N	N	N	N	N	N	N	N	N	N
Did the study provide estimates of random variability in the data analysis of relevant outcomes	NA	N	N	Y	Y	NA	Y	NA	Y	N	NA	NA	Y
Were adverse events reported?	Y	Y	Y	Y	Y	Y	Y	Y	Y	Y	Y	Y	Y
Were the conclusions of the study supported by the results	Y	Y	Y	Y	Y	Y	Y	Y	Y	Y	Y	Y	Y
Were both competing interest & sources of support for the study reported?	Y	Y	P	P	P	N	P	P	Y	Y	N	Y	Y
** N = No ** Y = Yes ** P = Partial ** U = Unclear ** NA = Non-applicable													

### Incidence & median time to bleed

The included literature reported a total of 470 cancer specific post-TORS bleeding events (4.77%) out of a total of 9852 TORS cases performed for malignant indications. The pooled average incidence of post-TORS haemorrhage across the 28 studies was found to occur in 7.24% (95% CI: 5.27–9.87; *I*^2^ = 89.6%) of HNC patients managed via TORS (range: 1.60–18.50%). A total of 4 patients suffered additional bleeding events, following their initial episode. The median time to an initial bleeding episode following TORS was found to be post-operative day 6 for HNC patients [23, 25, 27, 29, 33, 34, 40, 43, 45].

3.1 Severity.

A total of 7 papers specified severity of post-TORS bleed in HNC patients [22, 24, 27, 29, 30, 32, 33]. In 6/7 papers, bleeding episodes were classified using the Mayo Clinic classification system for transoral haemorrhage; this represented a total 22.3% of bleeds classified using the Mayo classification system [22]. One paper specified severity according to 2 different severity grading systems: Common Terminology Criteria for Adverse Events (CTCAE) & Hinni transoral surgery bleeding scale (HG) [30].

In total 28.5% of bleeds reported in this review were classified according to the aforementioned bleeding severity classification systems. It is important to highlight that the vast majority of bleeds reported across the 28 studies did not classify post-TORS haemorrhage severity (71.5%). The bleeding severity classification findings are summarised in Tables 5 and 6.

**Table 5:** Bleeding episodes incidence: classified using MAYO clinic classification system (Table 1)

	Mean	Range
Minor	3.0%	1.0–6.3.0.3%
Intermediate	2.72%	1.4–4.6%
Major	1.64%	1.0–2.2.0.2%
Severe	2.2%	0.7–3.8%

**Table 6:** Alternative bleeding severity classification systems

Grade	HG	CTCAE
	No. (bleed as % TORS cases)	No. (bleed as % TORS cases)
1	4 (1.9%)	13 (6.0%)
2	14 (6.3%)	1 (0.4%)
3	8 (3.7%)	14 (6.3%)
4	2 (1.0%)	1 (0.4%)
5	0	0
6	0	n/a
Total	28	29

** HG = Hinni transoral surgery bleeding scale

** CTCAE = Common Terminology Criteria for Adverse Events

### Mortality

The cause specific mortality for post-TORS haemorrhage in these studies was 0.43%.

### Bleed subsite

For the papers that documented the surgical site, the oropharynx was implicated in 97.06% of cases. The two main subsites were found to be the base of tongue (51.5%) and tonsil (45.6%) [25, 29, 33, 43, 45].

### Management of post TORS bleed for HNC patients

A total of 11 papers had complete data sets regarding the management of post-TORS haemorrhage. Out of the 102 patients represented in these papers, 72.5% were managed operatively, 23.5% managed conservatively with observation and 3.9% managed via interventional radiology (IR). The incidence of tracheostomy as part of airway management for post-TORS haemorrhage was 1.3%.

A total of 5 papers provided detailed descriptions for the management of 57 individual post-TORS bleeding events [23, 24, 29, 31, 34]. We chose to categorise interventions reported in these studies into two broad categories: procedural versus non-procedural. Procedural management represented all patients requiring either operative or interventional radiological management to achieve haemostasis, whereas non-procedural referred to patients managed largely through observation. Operative management was further divided into transoral haemostasis or transcervical haemostasis. Findings are summarised in Table 7. Transoral method of haemostasis was opted for in 77% of cases, with transcervical (5%), interventional radiology (5%) & observation (14%) accounting for the minority of haemostatic management strategies across these six studies. It is important to mention that in 4 cases no bleeding site was identified when transoral approach to haemostasis was opted for.

**Table 7:** Broad classification of approach to haemostasis following post-TORS bleed

Classification	Tools for haemostasis
Transoral (operative)	♣ Electrocautery: monopolar or bipolar diathermy ♣ Haemoclips ♣ Suture ligation
Transcervical (operative)	♣ Ligation of external carotid artery (ECA) and/or its branches
Interventional radiology (IR)	♣ Embolisation of external carotid artery (ECA) and/or its branches
Observational	♣ Haemostasis achieved without the need for return to theatre or interventional radiology input

### (i) predisposing factors associated with post-TORS haemorrhage

#### Antithrombotic medication usage

**Fig. 2:**
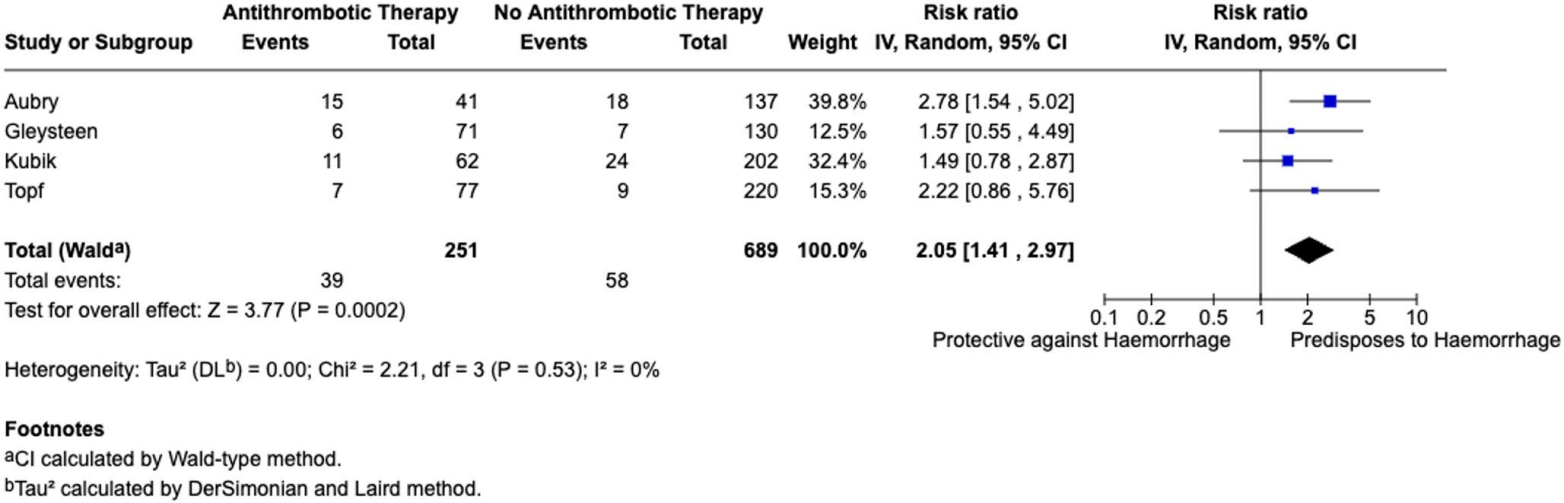
Meta-analysis of antithrombotic medication usage

A total of 4 studies assessed the impact of perioperative antithrombotic medication use on bleeding rates [23, 29, 33, 46]. The pooled post-TORS bleeding rate for patients receiving antithrombotic medication was 15.91% versus 8.12% for those not taking antithrombotic medication. Analysis found a significant increased risk of post-TORS haemorrhage with perioperative antithrombotic usage (RR = 2.05, 95% CI: 1.41–2.97; *I*^2^ = 0%). Findings are summarised in Fig. 2.

#### Prior irradiation

**Fig. 3:**
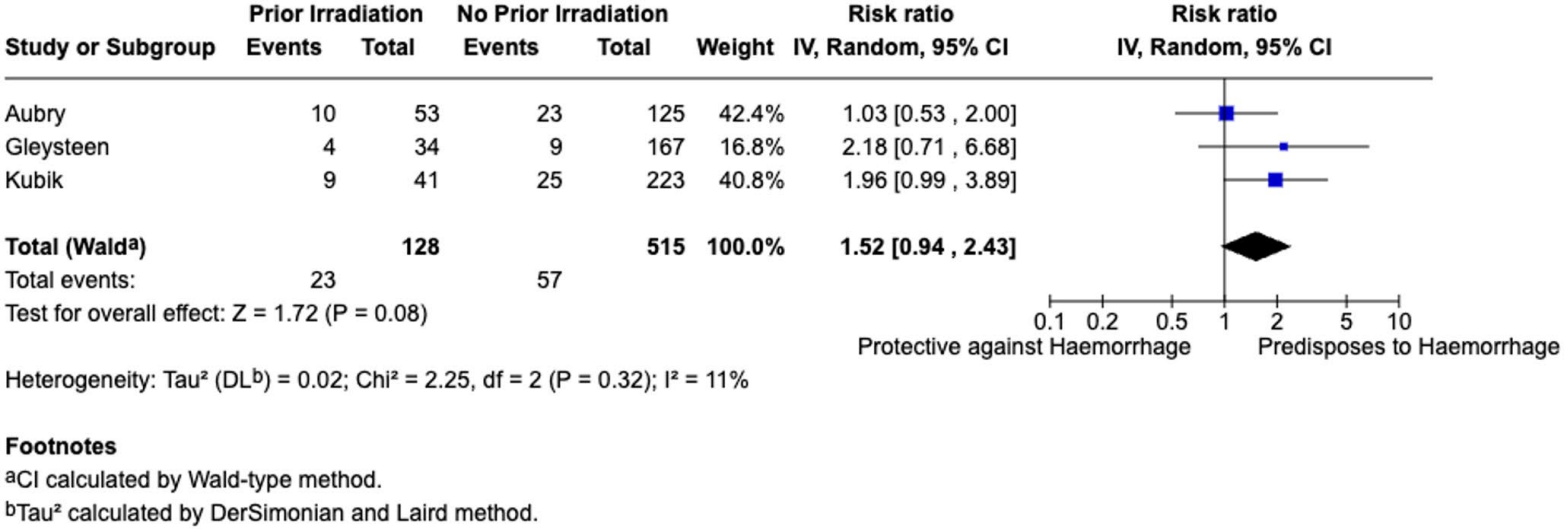
Meta-analysis of prior irradiationStage of disease

A total of three studies assessed impact of salvage surgery on post-TORS haemorrhage [23, 29, 33]. The pooled post-TORS bleeding rate was 18.36% for patients with irradiation prior to TORS versus without prior irradiation 10.89%. Prior irradiation was associated with a non-significant increased risk of post-TORS haemorrhage (RR = 1.52, 95% CI: 0.94–2.43; *I*^2^ = 11.0%). Findings are summarised in Fig. 3.

#### Stage of disease

**Fig. 4:**
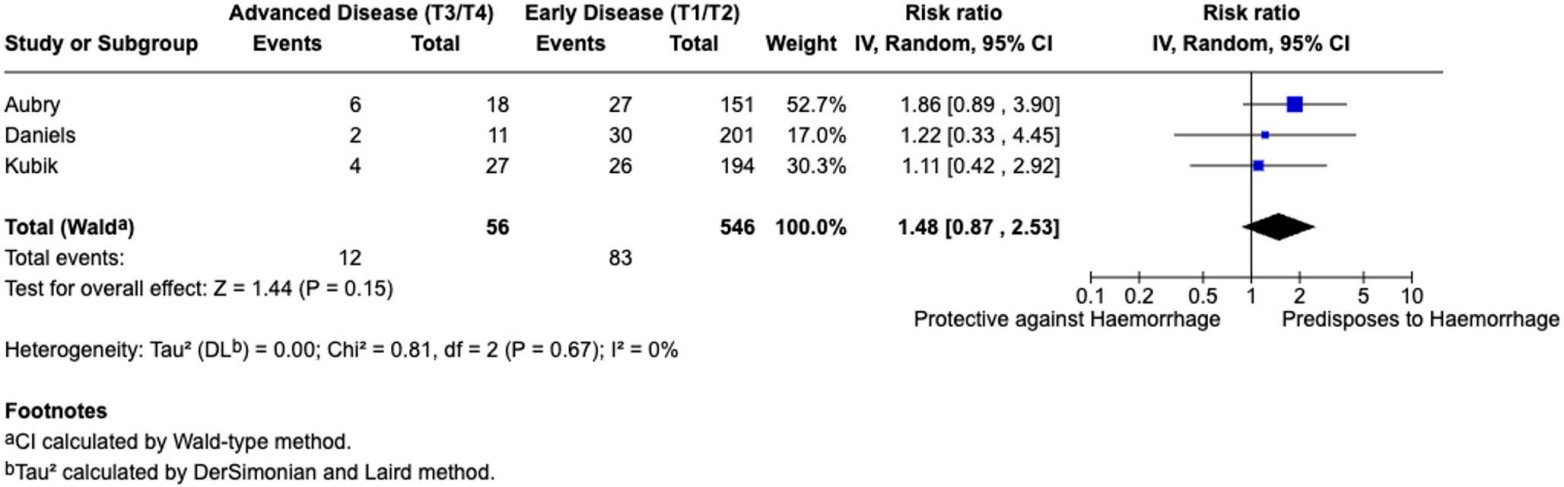
Meta-analysis of disease stage

In total three studies assessed impact of advanced (T3/T4) versus early (T1/T2) stage of cancer upon post-TORS haemorrhage rates [23, 27, 33]. The pooled bleeding rate for patients with advanced disease was 22.36% compared to 15.28% for early staged disease. Advanced disease stage was associated with increased risk of post-TORS haemorrhage but failed to reach significance in our analysis RR = 1.48, 95% CI: 0.87–2.53; *I*^2^ = 0%). Findings are summarised in Fig. 4.

#### Surgeon experience

**Fig. 5:**
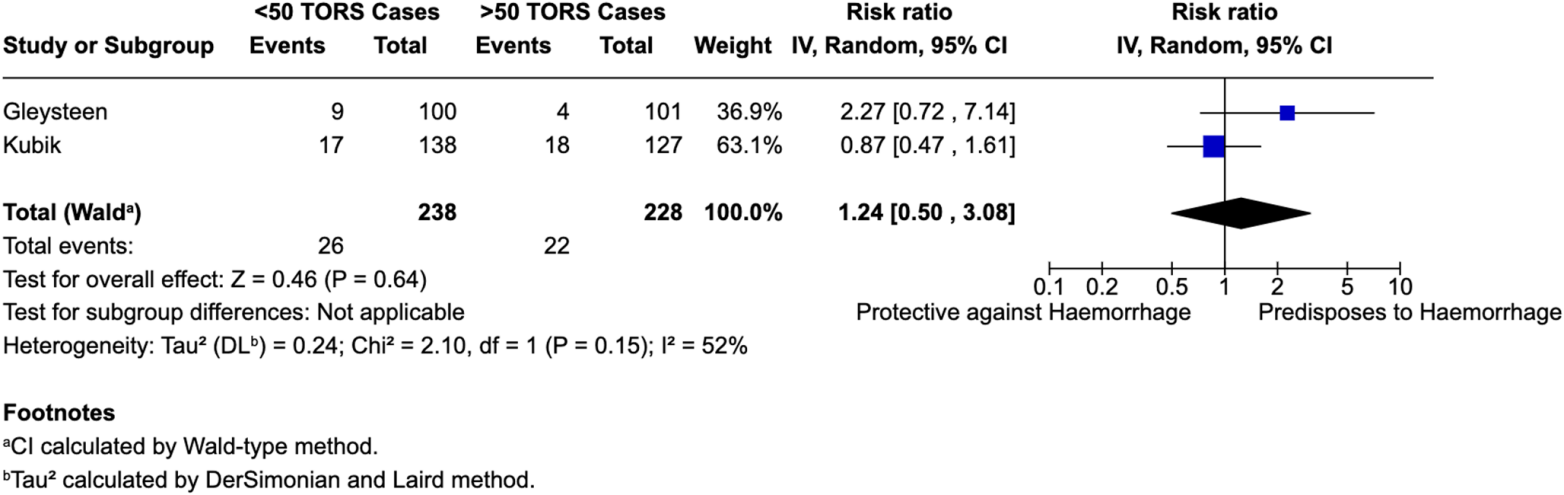
Meta-analysis of surgeon experience

Two papers reported the impact of surgeon experience, reflected by the number of TORS cases (< 50 or > 50), upon bleeding rates [29, 33]. A reduced level of surgeon experience (case experience < 50) was associated with a non-significant increased risk of bleeding following TORS (RR = 1.24, 95% CI: 0.50–3.06; *I*^2^ = 52.0%). Findings are summarised in Fig. 5.

### (ii) impact of prophylactic arterial ligation upon post-TORS haemorrhage

**Fig. 6:**
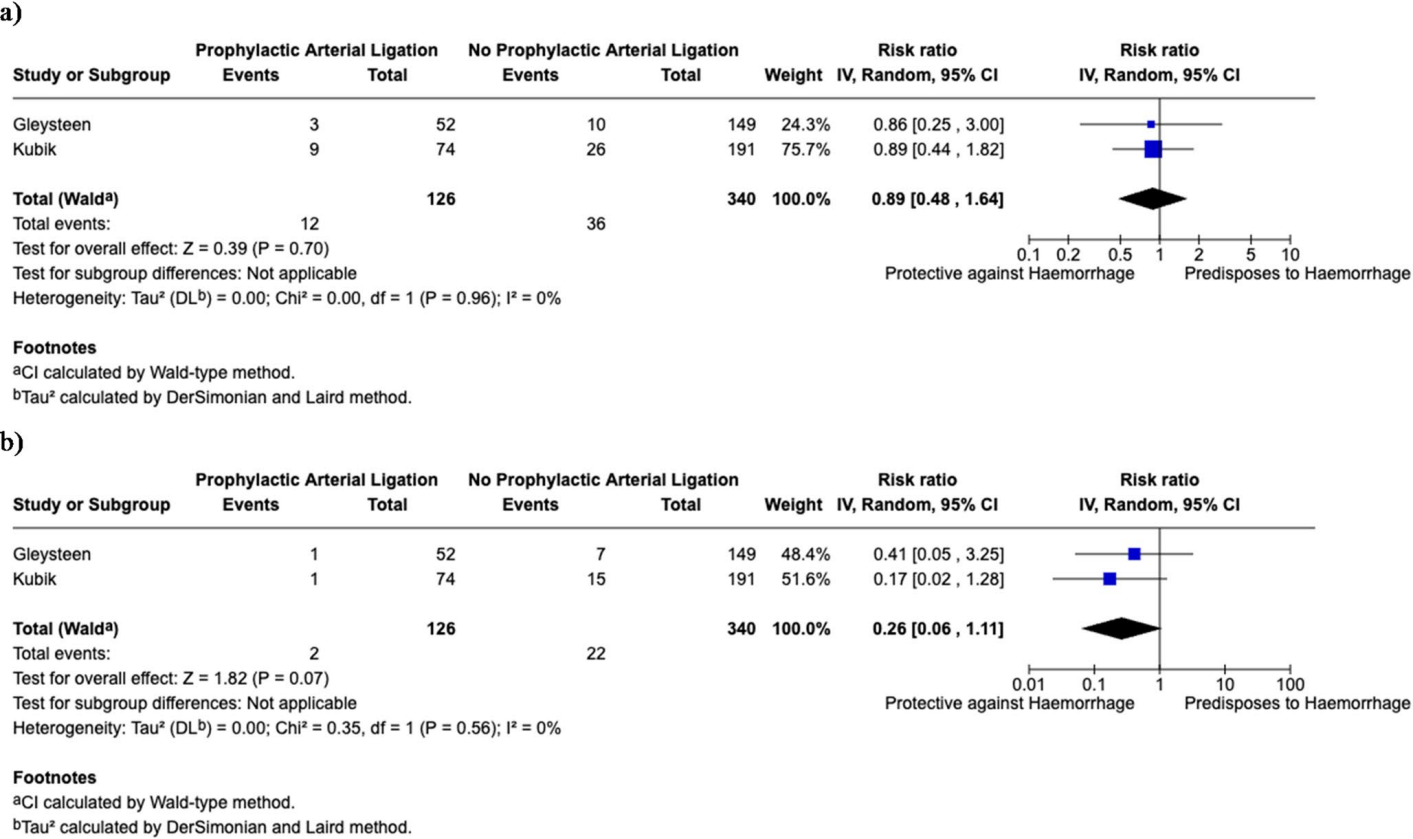
(**a**) Impact of prophylactic arterial ligation on post-TORS haemorrhage, (**b**) Impact of prophylactic arterial ligation on major/severe post-TORS haemorrhage

Two studies commented on the impact of prophylactic arterial ligation for preventing post-TORS bleeds [29, 33]. A total of 126 patients underwent prophylactic ligation versus 340 who did not. The pooled bleeding rate for patients with prophylactic ligation was 9.57% versus without 9.98%. Prophylactic ligation was found to represent non-significant reduction in risk (RR = 0.89, 95% CI: 0.48–1.64; *I*^2^ = 0%). Findings are summarised in Fig. 6a. It is important to note, that with respect to the impact of prophylactic ligation on severe/major post-TORS haemorrhage, prophylactic ligation is likely to be protective at reducing the risk of higher severity bleeds, however this did not reach significance (RR = 0.26, 95% CI: 0.06–1.11; *I*^2^ = 0%). The pooled major/severe haemorrhage rate for non-ligated patients was 6.50% versus 1.61% for patients who were prophylactically ligated prior to TORS. Findings are summarised in Fig. 6b.

## Discussion

Haemorrhage following TORS represent the most severe complication associated with TORS, with a pooled bleeding incidence for HNC patients of 7.24% (1.60–18.50%) [10, 22–48]. This review provides an overview of the bleeding rates, severity & management strategies employed specifically in head and neck cancer patients experiencing post-TORS haemorrhage reported in the literature over the last 12 years (2013–2025).

As the utilisation of TORS for benign and malignant indications increases, the risk of post-TORS haemorrhage and potential subsequent risk of airway compromise becomes a more pressing issue, especially for large volume centres, performing multiple TORS cases per week. It is therefore vital to identify patients who possess predisposing risk factors which may increase the risk of post-TORS haemorrhage, during pre-operative surgical assessment, to help inform decisions regarding, length of stay in hospital, reintroduction of antithrombotic therapy and need for prophylactic vessel ligation.

Our meta-analysis found that antithrombotic therapy use was associated with a significant increased risk of post-TORS haemorrhage (RR = 2.05, 95% CI: 1.41–2.97). It is important to highlight that majority of post-TORS haemorrhages present in the included literature took place at a time (i.e. median day of bleed, POD 6) far later than most guidelines would recommend resuming antithrombotic therapy [49, 50]. Critically, protocol driven re-initiation of antithrombotic therapy must be assessed on an individual basis, to ensure a risk-benefit approach is taken to mitigate the risk of post-TORS haemorrhage when resuming antithrombotic therapy were possible. Identification of this potential risk factor in pre-operative assessment may serve to better guide selection of the post-operative recovery setting and the extent of clinician monitoring required for patients; to enable prompt intervention should these patients suffer a post-operative bleed. Furthermore, there was a paucity of information with respect to the specific drug and dosage associated with post-TORS haemorrhage, therefore limiting conclusions regarding specific causative antithrombotic agents. Continued research is needed to guide specific recommendations.

History of prior irradiation was found to closely approach statistical significance as a predisposing risk factor for post-TORS bleeds (RR = 1.52, 95% CI: 0.94–2.43). This is likely due to local changes associated with prior irradiation, including delayed healing and increased fibrosis, which complicates the surgical procedure and post-operative recovery further [29, 51]. Intraoperatively, as with open procedures, tissue planes are often not as well demarcated which leads to more tissue dissection and increased energy delivery to the tissues, both of which are known to increase the risk of post-operative haemorrhage.

Advanced disease stage (T3/T4) also showed a trend suggesting an increased risk of post-TORS haemorrhage (22.36% versus 15.28%), but did not achieve significance in our analysis (RR = 1.48, 95% CI: 0.87–2.53). This increased risk is likely due to the extent of invasion associated with advanced stage tumours, leading to distorted anatomy and an increased degree of difficulty with dissection during TORS procedures.

Surgeon experience was not found to be associated with a significant increased risk of post-TORS haemorrhage. This, however, was not supported by Chia et al. [52] who noted a significant decrease in complications associated with higher surgeon case volume. To fully assess the impact of surgeon experience, case matched examples would need to be compared as often more experienced surgeons are taking on more complex cases. From this review we are able to conclude that if there is a threshold of caseload to see a reduction in post-operative haemorrhage it is likely less than 50 cases.

The ECOG 3311 trial (NCT01898494) demonstrated a reduced rate of major and severe bleeding when prophylactic ligation was performed [53]. Our analysis suggested that there was no significant reduction in overall bleeding rates between patients undergoing prophylactic arterial ligation versus those who did not (RR = 0.89, 95% CI: 0.48–1.64). Furthermore, prophylactic ligation is likely to be protective at reducing the risk of severe bleeding events, however this did not achieve significance in our review (RR = 0.26, 95% CI: 0.06–1.11).

Our analysis found that severity of post-TORS bleeds were classified using 3 different classification systems. The most widely accepted in clinical practice and the most widely utilised in these studies was the Mayo Clinic classification system (22.3%). Overall, haemorrhage severity was classified for less than a third (28.5%) of the total bleeding events in the included literature. Without standardised severity classification of post-TORS haemorrhages, the comparison of management approaches for bleeds of differing severity across different centres becomes challenging.

Currently, a unanimous approach regarding an appropriate protocol for management of post-TORS bleeding events does not exist. This is illustrated by findings in Table 7, which demonstrates the varied nature of procedural and non-procedural interventions employed across a range of institutions, as reported by the included literature. Out of the 5 papers that provided granularity with respect to haemostatic techniques, a transoral approach was opted for in 77% of cases. This involved a combination of electrocautery, suture ligation and haemoclips, however, the utilisation of these tools for achieving haemostasis varied between different [23, 24, 29, 31, 34].

It is important to highlight that whilst the pooled average bleeding incidence (7.24%) provides a summary across the studies, the substantial heterogeneity (*I*^*2*^ = 89.6%) suggest that the studies may not all be estimating the same effect and therefore this pooled estimate should be interpreted with caution; as the clinical applicability of this finding may be context dependent. Furthermore, a moderate degree of heterogeneity was found when assessing the impact of surgeon experience on post-TORS haemorrhage rate (*I*^*2*^ = 52.0%). Given the limited number of studies, further research is needed to clarify whether surgical volume meaningfully influences bleeding risk.

Findings from this systematic review must be interpreted in the context of its limitations. This review encompasses 12 years’ worth of reported surgical literature pertaining to post-TORS haemorrhage; including papers with a large degree of variability in the year and duration of study periods in which TORS haemorrhage was assessed. This presents a distinct challenge when it comes to drawing conclusions with respect to post-TORS haemorrhage, as studies reporting TORS outcome data closer to the 2009 FDA approval for malignant indications, arguably lack the breadth of knowledge which surgeons of present day possess, which may subsequently have impacted the incidence of post-TORS haemorrhage reported. Furthermore, the largely retrospective non-randomised nature of the included studies, served to limit the conclusions that could be drawn, when comparing utilisation of one haemostatic technique over another. Finally, there was significant variability in the reporting of data noted across the 28 studies, especially for data specifying techniques for achieving haemostasis. The heterogeneity in management of bleeding events further emphasises the need for consensus and a universally supported approach amongst head and neck cancer surgeons for achieving haemostasis and reducing associated morbidity and mortality.

## Conclusion

Post-TORS haemorrhage represents the most severe complication associated with TORS. Factors associated with an increased risk of post-TORS haemorrhage were assessed in the meta-analysis portion of this review, to help consolidate the existing knowledge base. The varied nature of haemostatic management techniques employed in the included literature, indicates the need for the surgical community to work towards a standardised system for documenting details relating to the post-TORS haemorrhage and techniques for achieving haemostasis. This would support a more robust comparison of the outcomes from this uncommon but potentially fatal complication.

## Data Availability

No datasets were generated or analysed during the current study.

## Appendix

### Appendix 1: Search strategy

Transoral robotic surgery OR trans-oral robotic surgery OR TORS OR robotic transoral approach.

AND

Haemorrhage OR hemorrhage OR bleeding.

AND

Head and neck.

### Appendix 2: PICO framework

**Population**

Inclusion: Adults (>18 years old) with head and neck cancer, treated using transoral robotic surgery (TORS)

**Exclusion:**

- Patients < 18 years old.
- Patients > 18 years old without head and neck cancer
- Patients > 18 years old with head and neck cancer, treated without TORS

**Intervention(s) or exposure(s)**

Inclusion criteria: Post-TORS haemorrhage in head and neck cancer patients

Exclusion criteria: Post-TORS haemorrhage for indications other than malignancy

**Comparator(s) or control(s)**

Not applicable

**Outcomes**

- Incidence of post-TORS haemorrhage in head & neck cancer patients
- Severity of post-TORS haemorrhage

**Additional outcomes**

- Predisposing risk factors associated with post-TORS haemorrhage
- Method of management for post-TORS haemorrhage (conservative versus surgical)
- Rebleeding episodes following initial post-TORS haemorrhage
- Length of stay following post-TORS haemorrhage
- Patient demographics

**Timeframe**

Post-TORS haemorrhage episode occurring within 14-days following TORS

**Study type**

Observational study designs, including case-control studies, cohort studies & case series were included.
